# Niche breadth and niche overlap of typical plant communities in the plateau temperate semi-arid river valley climatic region of the Qinghai-Xizang plateau

**DOI:** 10.3389/fpls.2026.1749810

**Published:** 2026-04-09

**Authors:** Benyi Fu, Wei Yang, Jie Yang, Daqing Luo, Xingle Qu

**Affiliations:** 1 National Forest Ecosystem Observation & Research Station of Linzhi Xizang, Xizang, Linzhi, China; 2 Institute of Plateau Ecology, XiZang Agricultural and Animal Husbandry University, Linzhi, China; 3 Key Laboratory of Alpine Vegetation Ecological Security of Husbandry University, Linzhi, China; 4 Key Laboratory of Tibet Plateau Forest Ecology of Ministry of Education & National, Linzhi, China

**Keywords:** Alpine semi-arid river valleys, community stability, herbaceous layer, interspecific associations, niche characteristics, Qinghai-Xizang plateau

## Abstract

**Introduction:**

The alpine semi-arid river valleys of the eastern Qinghai-Xizang Plateau are characterized by strong environmental heterogeneity and ecological fragility. Herbaceous plants dominate these ecosystems; however, integrative studies linking niche differentiation, interspecific associations, and community stability remain limited, particularly under extreme alpine conditions.

**Methods:**

Based on consecutive field surveys conducted from 2021 to 2025 in Qamdo City, we investigated 109 sampling plots comprising 327 herbaceous quadrats. We quantified niche breadth and niche overlap of dominant species and analyzed interspecific associations and community stability using classical ecological indices and statistical approaches.

**Results:**

Dominant species showed clear differentiation between broad-niche generalists and narrow-niche specialists (Levins: 1.03–19.18; Shannon: 0.67–3.15). Overall niche overlap was low, with 70% of values within 0–0.5, indicating strong resource-use differentiation. The herb layer exhibited a weak and non-significant positive overall interspecific association. Significant positive associations mainly occurred among broad-niche species, whereas negative associations were concentrated among species with similar resource requirements. Community stability was insufficient, with a Euclidean distance of 17.22 from the theoretical stable point, suggesting limited structural and functional resilience.

**Discussion:**

Pronounced niche differentiation and generally weak interspecific associations represent key adaptive strategies of herbaceous communities in this alpine semi-arid valley system. These patterns reduce competitive exclusion but may also constrain overall community stability. The findings provide important ecological insights and a scientific basis for biodiversity conservation and ecosystem restoration on the Qinghai-Xizang Plateau.

## 1 Introduction

Niche is a core ecological concept defining a species’ resource acquisition, utilization strategies and environmental adaptability, which underpins the mechanistic understanding of species coexistence (Grinnell, 1917; Hutchinson, 1957; Nan et al., 2022). Its multi-dimensional attributes (spatial distribution, resource use breadth, environmental tolerance) directly determine a species’ survival potential and competitive ability in heterogeneous habitats (Wu et al., 2024). Interspecific associations, describing the spatial co-occurrence and interdependence between species, are direct manifestations of species interactions: positive and negative associations respectively reflect facilitative and competitive relationships shaped by niche differentiation and resource demand (Xiang et al., 2024; Lin et al., 2025), and are fundamental for deciphering community assembly rules and predicting dynamics under environmental change (Jin et al., 2025). Community stability refers to an ecosystem’s capacity to resist external disturbances, maintain structural and functional integrity, and recover after perturbation, serving as a comprehensive metric of ecosystem health (Hou et al., 2023; Liu et al., 2025). Classic and contemporary ecological studies have demonstrated that community stability is jointly regulated by species-level niche adaptation and community-level interspecific interactions: niche differentiation alleviates competitive exclusion and enhances disturbance resistance, while positive interspecific associations facilitate species coexistence and improve community resilience (McCann, 2000; Tilman, 2004; Isbell et al., 2015; Chen et al., 2023). Accordingly, niche characteristics, interspecific associations and community stability form a logically linked, indivisible research framework for unraveling plant community assembly—from individual species’ resource use strategies to interspecific interaction patterns, and ultimately to the steady-state maintenance of the whole community. The expression and regulatory mechanisms of these three linked attributes under extreme environmental conditions remain a central focus of global ecological research (Kim and Ohr, 2020; Wang et al., 2022).

The Qinghai-Xizang Plateau, with an average elevation over 4000 m, is Earth’s highest plateau with unique ecological attributes, playing an irreplaceable role in global climate regulation, water security and biodiversity conservation, while also being a sensitive and fragile region under global climate change (Dong et al., 2023; Zhao et al., 2025). The alpine semi-arid river valley system, a representative geomorphic and ecological unit in the eastern Qinghai-Xizang Plateau, is an integrated ecological complex formed by long-term fluvial and tectonic processes, rather than a simple juxtaposition of isolated habitat patches (Wu et al., 2024; Jin et al., 2025). Along its continuous “riverbed–floodplain–terrace–valley slope” spatial sequence, hydrology, water availability, temperature and soil physicochemical properties form a tightly coupled, continuously changing environmental gradient (Liu et al., 2025). Under the harsh alpine environment (low temperature, hypoxia, strong ultraviolet radiation, seasonal drought), constrained by drought, low temperature and poor soil nutrients, woody plants only occur sporadically in favorable microhabitats. In contrast, herbaceous plants, with flexible reproductive strategies, short growth cycles and strong environmental tolerance, are widely distributed across all habitat types and constitute the absolute dominant component of the valley vegetation system (Matsuo et al., 2024). Their niche differentiation, interspecific interactions and community dynamics directly shape the structural stability and functional performance of the alpine semi-arid valley ecosystem, and are key indicators of valley ecosystem health under climate change (Lin et al., 2025; Jin et al., 2025). Notably, the ecological integrity of this valley system does not exclude environmental heterogeneity; instead, the continuous gradient heterogeneity is integrated into a synergistically operating unified ecological framework (Zhang et al., 2018; Dunham et al., 2024). The river channel acts as the core hydrological hub, forming a continuous moisture gradient through runoff, infiltration and lateral recharge; synchronous continuous gradients of microclimate and soil properties also develop along the spatial sequence, collectively forming the integrated environmental matrix for herbaceous plant survival and colonization (Lin et al., 2016; Wu et al., 2024; Liu et al., 2025; Xiang et al., 2025). Within this integrated habitat, the ecological processes of herbaceous communities show strong spatial coherence: both broad-niche generalists and narrow-niche specialists exhibit integrated adaptive responses to the whole valley environmental system, and interspecific associations are driven by the region-wide continuous resource distribution and environmental pressure, rather than isolated habitat patches (Nan et al., 2022; Lin et al., 2025; Xiang et al., 2024; Zhao et al., 2025). This confirms that only a whole-system perspective can effectively reveal the niche differentiation, interspecific association and community stability mechanisms of the valley herb layer (Jin et al., 2025; Liu et al., 2025).

Qamdo City, located in the eastern Qinghai-Xizang Plateau in the core area of the Hengduan Mountains, is the core distribution area of typical alpine semi-arid river valley systems, covering the upper reaches of the Lancang River, Jinsha River, Nujiang River and their major tributaries. The continuous spatial sequence of valley sub-geomorphic units here forms a unified environmental gradient and integrated habitat for herbaceous vegetation, making the dominant herb layer an ideal model for exploring alpine plant community assembly rules and stability maintenance mechanisms. However, due to high altitude, harsh natural conditions, poor accessibility and wide continuous environmental gradients, systematic and integrative studies on herbaceous communities in this region are severely scarce. Existing studies have not yet incorporated niche differentiation, interspecific associations and community stability into a unified analytical framework for this alpine semi-arid river valley system, leading to an unclear mechanistic understanding of herbaceous plant adaptive strategies and community steady-state maintenance in this extreme heterogeneous habitat. Based on classic niche theory, community assembly theory and global research progress on alpine plant communities, we propose three core testable research hypotheses to fill the above knowledge gaps:The continuous environmental heterogeneity in the alpine semi-arid river valley system drives significant differentiation in niche breadth among dominant herbaceous species, forming a clear divergence between broad-niche generalist species and narrow-niche specialist species. Meanwhile, the overall niche overlap among species is at a low level, and species achieve stable coexistence through fine-scale partitioning of multi-dimensional resources, thus avoiding competitive exclusion.The interspecific association pattern of dominant herbaceous species is directly shaped by their niche characteristics. The overall interspecific association of the herbaceous layer presents a weak positive correlation that is not statistically significant; significant positive associations mainly occur among broad-niche generalist species with complementary resource use strategies, while significant negative associations are concentrated among species with high similarity in resource requirements and high niche overlap.The stability of the herbaceous community in the study area is synergistically regulated by species niche characteristics and interspecific associations. The community will show insufficient overall stability, which is mainly constrained by the dispersed dominance of dominant species, the limited number of broad-niche species, and the generally weak interspecific associations; meanwhile, the pronounced niche differentiation will endow the community with a certain degree of disturbance resistance potential.Based on systematic field surveys and laboratory analyses across Qamdo City, this study focuses on herbaceous communities in the alpine semi-arid river valley systems of the eastern Qinghai-Xizang Plateau, and aims to test the above hypotheses and address the critical research gaps through three specific objectives: (1) Quantify the niche breadth and overlap of dominant herbaceous species, clarify their resource-use strategies and niche differentiation patterns, and identify generalist and specialist species in the community; (2) Analyze the overall interspecific association patterns among dominant species, reveal the dominant type of species interactions, and explore the relationship between niche characteristics and interspecific associations; (3) Evaluate the stability level of herbaceous communities, and elucidate the joint regulatory mechanism of niche differentiation and interspecific associations on community stability. This study systematically reveals the adaptive strategies and community stability maintenance mechanisms of herbaceous communities in the target alpine semi-arid valley ecosystems, filling key knowledge gaps in the ecological research of semi-arid valley herb layers in the eastern Qinghai-Xizang Plateau. The findings enrich the theoretical understanding of alpine plant community assembly in extreme arid environments, and provide a solid scientific basis and practical guidance for vegetation conservation, ecological restoration and sustainable management of alpine semi-arid valley ecosystems on the Qinghai-Xizang Plateau under global climate change.

## 2 Materials and methods

### 2.1 Overview study area

**Figure 1:**
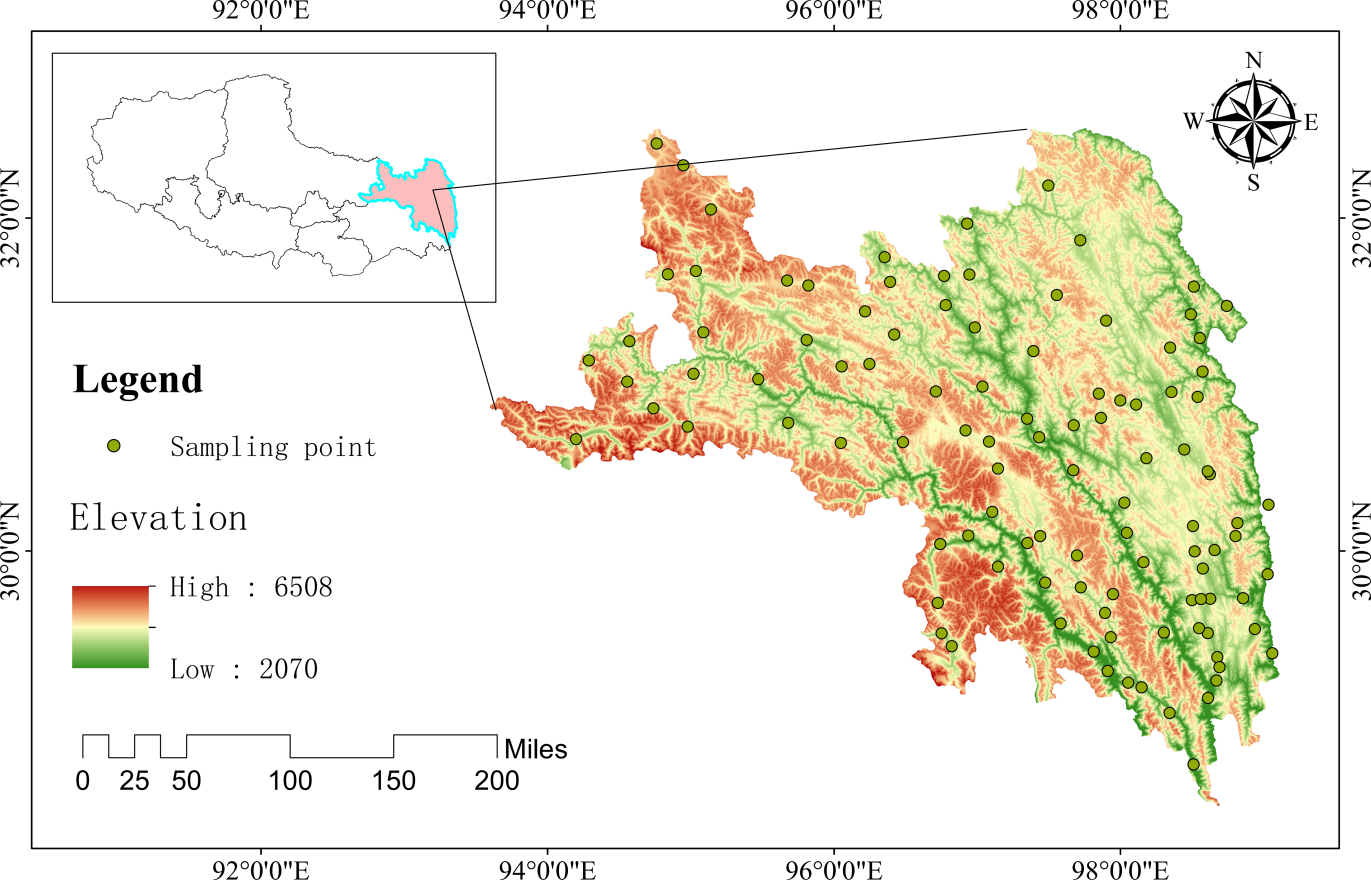
Geographic location of Qamdo City in the eastern Qinghai-Xizang Plateau and distribution of herbaceous community sampling plots.

The study area is located in Qamdo City, southeastern Qinghai-Xizang Plateau, in the northern section of the Hengduan Mountains (core area of the upper reaches of the Three Parallel Rivers: Jinsha, Lancang and Nujiang Rivers), with a geographic range of 93°06′ E–99°02′ E, 28°05′ N–32°06′ N. It covers the upper and middle reaches of the three rivers and their major tributaries, dominated by alpine gorge landforms with dramatic topographic relief and large vertical elevation differences.This region is characterized by a plateau temperate semi-arid monsoon climate with significant vertical zonal differentiation, jointly regulated by the southwest, southeast and plateau monsoons; local plateau temperate semi-humid to humid monsoon climates occur in high-elevation eastern and southern mountain areas due to orographic lifting. The climate features low annual average temperature with remarkable diurnal variation (extreme minimum winter temperature of –16 °C to –22 °C), cool summers without sustained high temperature, and a significant vertical decrease in temperature with rising elevation. Precipitation shows strong seasonal and spatial heterogeneity: over 90% of annual precipitation is concentrated in May–September, with 400–550 mm in the central dry valley zone, 800–1200 mm in high-elevation mountain areas, dominated by nocturnal rainfall. The region also has high solar radiation and strong ultraviolet intensity, with prevailing westerly winds in winter and spring, and fewer strong wind days than the western Qinghai-Xizang Plateau due to the topographic barrier of the Hengduan Mountains.The continuous hydrothermal, topographic and habitat gradients in this area provide a representative natural experimental system for exploring the niche differentiation, interspecific associations and community stability of alpine valley herbaceous communities (Wang et al., 2016; Ciwang et al., 2025) (**Figure 1**).

### 2.2 Research methods

We established sampling plots across the alpine semi-arid valleys of the study area during the peak growing season of alpine herbaceous plants in 2021. Plot layout was designed based on high-resolution remote sensing imagery and systematic field reconnaissance, to ensure full coverage of all representative herbaceous habitat types along the valley habitat gradient. From 2022 to 2025, we conducted annual fixed-position repeat surveys of all plots in the same peak growing season, to verify the temporal stability of herb layer species composition, minimize random errors from single-year surveys, and ensure the reliability of the dataset for subsequent analyses.For each plot, a 50-m linear transect was set up along the dominant direction of the valley habitat gradient (from riverbed to valley slope). Three 1 m × 1 m herbaceous quadrats were systematically arranged along each transect (at 20 m, 40 m and the 50 m endpoint), yielding 3 quadrats per plot. In total, 109 independent sampling plots and 327 corresponding quadrats were established across the study area.For each quadrat, we recorded geographic information (longitude, latitude, elevation) via high-precision handheld GPS, and measured key herb layer community traits, including the full scientific name of each vascular plant, individual abundance, average plant height, and canopy coverage. Species unidentified *in situ* were collected as voucher specimens with unique plot labels and photographed for diagnostic morphological traits; all specimens were deposited in a certified professional herbarium, with final identification completed by senior plant taxonomists to ensure taxonomic consistency across the survey period.Plots subjected to severe anthropogenic disturbance (intensive overgrazing, engineering construction) or extreme natural disturbance (flood inundation, debris flow) were excluded from analyses to avoid confounding effects on community structure and niche metrics. No abrupt extreme climate events that would significantly alter species richness and community composition were recorded during the 5-year monitoring period, and the large sample size of fixed-position monitoring minimized confounding effects of short-term community succession, ensuring we captured the stable core characteristics of the herbaceous communities.

### 2.3 Data statistical analysis

#### 2.3.1 Data preprocessing and dominant species selection

No significant interannual fluctuation in species quantity was observed during the 5-year field survey. We calculated the important value (IV) of each species for each survey year, then used the 5-year average IV of each species per quadrat for subsequent analyses. Following the standard dominant species screening protocol (Zhang et al., 2018; Keddy, 2007), we selected the top 20 species (IV accounting for >1% of the total IV) as dominant species for further analysis of niche traits and interspecific associations.

#### 2.3.2 Niche metrics calculation

We adopted four classic niche metrics for analysis, with all indices calculated based on the proportion of each species in the quadrats (total number of quadrats as the total number of resource states):Levins niche breadth index (Levins, 1968) and Shannon diversity index (Silvertown, 2015) to quantify the resource utilization range and habitat adaptability of each species; Pianka niche overlap index (Pianka, 1978) to measure the degree of resource utilization overlap between species pairs;Schoener niche similarity index (Schoener, 1974) to characterize the similarity of ecological strategies between species.

#### 2.3.3 Interspecific association analysis

We first calculated the arithmetic mean of the 5-year abundance data of each species per quadrat to construct a species-quadrat abundance matrix for interspecific association and community stability analyses.Overall interspecific association: We used the variance ratio method (Schluter, 1978) to calculate the connectivity index (*VR*) to determine the direction and strength of the overall interspecific association (*VR*>1 indicates positive overall association, *VR* < 1 indicates negative overall association). We further used the connectivity strength statistic W to test the significance of the association: a W value outside the range of χ²_0.95_(N) to χ²_0.05_(N) indicates a statistically significant overall association.Pairwise species association: To reduce potential errors caused by small sample sizes or sparse data in 2×2 contingency tables (**Table 1**), we used the AC association coefficient and Ochiai (O*I*) index to quantify the strength of pairwise interspecific associations.

**Table 1:** Analysis of the 2×2 table for interspecific association.

	Species B is present	Species B is absent	Row total (number of plots)
Species B is present	*a*	*b*	*a*+*b*
Species B is absent	*c*	*d*	*c*+*d*
Column total (number of quadrats)	*a*+*c*	*b*+*d*	*N*=*a*+*b*+*c*+*d*

*a*, number of plots where both species occur; *b*=number of plots with only Species B; *c*, number of plots with only Species A; *d*=number of plots where neither species occurs; *N*, total sample size.

The *AC* coefficient ranges from -1 to 1: values approaching 1 indicate strong positive association, values approaching -1 indicate significant negative association, and a value of 0 indicates complete spatial independence between species (Hu et al., 2024). The OI index ranges strictly from 0 to 1: values closer to 1 represent stronger positive association and higher co-occurrence probability, while values closer to 0 reflect stronger spatial segregation and competitive exclusion (Guo et al., 2015).

### 2.4 Community stability analysis

In the study of plant community stability, the study area has a temperate climate, and the M. Godron method was used for evaluation (Zeng et al., 2025).The study selected the top 20 species ranked by importance value. According to this method, the relationship between the percentage of the reciprocal of the number of species and the corresponding cumulative relative frequency was analyzed, and a smooth curve model was constructed. By intersecting with the straight line *y* = 100 -*x*, the stability ratio of the community was obtained. And the closer this ratio is to the ideal stable point (20, 80), the higher the community stability.

The ecological niche width and niche overlap index were calculated using the spaa package in R 4.4.1. The *niche*() function was used to calculate the species’ niche width, *niche.overlap*() was used to assess niche overlap between species, sp.*assoc*() was used to obtain the connectivity coefficient AC, and the *cor*() function in the stats package was used to calculate the Ochiai correlation coefficient. Community stability was analyzed using MATLAB R2024b, and linear regression for analysis and plotting was performed using Origin 2025 Pro.

## 3 Results

### 3.1 Species composition of the herb layer in the study area

The field survey in Qamdo City recorded a total of 296 herbaceous plant species, belonging to 61 families and 167 genera. Among them, 23 species (16 genera, 7 families) are monocotyledons, and 241 species (142 genera, 46 families) are dicotyledons. The dominant families of the flora are Asteraceae, Poaceae, Fabaceae and Rosaceae. Constrained by the extreme alpine semi-arid environment of the study area, tree species are extremely rare, and shrubs are only distributed in patchy microhabitats, while herbaceous plants constitute the absolute dominant component of the regional vegetation. Accordingly, this study focuses on the herbaceous layer to analyze the resource utilization patterns of plant species in the alpine semi-arid valley ecosystems.

### 3.2 Analysis of niche characteristics

#### 3.2.1 Analysis of niche indices

The niche breadth of dominant herbaceous species showed significant differentiation (**Table 2**). The Levins niche breadth index ranged from 1.03 to 19.18, and the Shannon index ranged from 0.67 to 3.15, with highly consistent variation trends between the two indices. *Stipa purpurea* had the highest Levins index (19.18) and a top-tier Shannon index (2.73), followed by *Stipa subsessiliflora*, indicating these two species had the widest resource utilization spectrum and strongest adaptability to heterogeneous habitats. In contrast, *Oxytropis microphylla* had the lowest Levins (1.03) and Shannon (0.67) indices, followed by *Carex praeclara* and *Carex sagaensis*, reflecting extremely narrow resource ranges and high habitat specialization.

**Table 2:** Niche Breadth - related Indices of dominant species in herb layers.

Abbreviation	Plant name	Relative abundance	importance value	Levins	Shannon
SP	*Stipa purpurea* (Griseb)	0.14	14.51	19.18	2.73
CM	*Carex moorcroftii* Falc. ex Boott.	0.08	7.63	10.34	1.75
KP	*Kobresia pygmaea*	0.17	4.98	7.58	1.74
PC	*Ptilotricum canescens*(DC.)	0.01	4.55	5.95	1.90
OT	*Orinus thoroldii*	0.03	4.06	6.05	2.77
AW	*Artemisia wellbyi*	0.01	3.81	7.54	2.04
PE	*Pennisetum centrasiaticum*	0.02	3.77	1.98	1.55
PF	*Pennisetum flaccidum Griseb.*	0.01	3.67	1.91	1.48
FR	*Festuca rubra L.*	0.01	3.38	6.08	1.97
DC	*Dysphania schraderiana*	0.01	3.25	2.20	0.85
KR	*Kobresia royleana*	0.04	2.96	2.11	1.42
KM	*Kobresia macrantha*	0.07	2.12	1.74	1.75
AY	*Artemisia younghusbandii*	0.01	2.01	3.50	1.20
SS	*Stipa subsessiliflora (Rupr.)*	0.04	1.26	17.60	3.15
FO	*Festuca ovina*	0.02	1.64	3.47	1.73
AS	*Astragalus strictus*	0.01	1.50	2.10	0.80
CS	*Carex sagaensi*s	0.01	1.48	1.30	0.75
PS	*Polygonum sibiricum Laxm.*	0.04	1.40	3.28	1.60
CP	*Carex praeclara*	0.01	1.35	1.25	0.72
OM	*Oxytropis microphylla*	0.01	1.28	1.03	0.67

The abbreviations of species’ Latin names in the table within the image shall apply hereinafter.

Relative abundance showed a complex non-linear correlation with niche breadth. *Kobresia pygmaea* had the highest relative abundance (0.17) but only moderate niche breadth (Levins: 7.58, Shannon: 1.74), indicating strong resource specificity and high dependence on specific microhabitats despite its numerical dominance. *S. purpurea* (second-highest relative abundance, 0.14) presented a synergistic “high relative abundance - broad niche” pattern, maintaining population dominance via both quantitative superiority and extensive resource utilization capacity. *Kobresia macrantha* (relative abundance: 0.07, 4th among dominant species) also showed a “high abundance - narrow niche” pattern, while *S. subsessiliflora* had low relative abundance (0.04) but top-tier niche width, reflecting strong adaptive potential to diverse habitats despite limited population size. *Carex moorcroftii*, *Orinus thoroldii*, *Artemisia wellbyi* and *Festuca rubra* showed moderate, coordinated levels in both relative abundance and niche breadth, while *Pennisetum centrasiaticum*, *Pennisetum flaccidum* and *Astragalus strictus* had low values for both indicators, implying limited resource utilization and population expansion potential.

The importance value (IV), a comprehensive metric of species ecological dominance, had a more significant synergistic relationship with niche breadth. *S. purpurea* (highest IV: 14.51) and *C. moorcroftii* (second-highest IV: 7.63) showed a typical “high IV - broad niche” pattern, acting as the core dominant and sub-dominant species in the community, respectively. *K. pygmaea* (IV: 4.98) and *Ptilotricum canescens* (IV: 4.55) presented a “high IV - moderate/narrow niche” characteristic, with their dominant position dependent on population quantitative superiority and habitat-specific adaptation rather than extensive resource utilization. Notably, *S. subsessiliflora* had a low IV (1.26) but niche width close to *S. purpurea*, indicating extremely strong potential ecological adaptability, which may be limited by habitat availability, interspecific competition or environmental filtering rather than resource utilization capacity. *O. thoroldii*, *A. wellbyi* and *F. rubra* maintained stable community status via balanced moderate levels of IV and niche breadth, while species with low IV generally had extremely narrow niche widths, with minimal influence on community structure.

### 3.3 Correlation analysis amongniche indices

**Figure 2:**
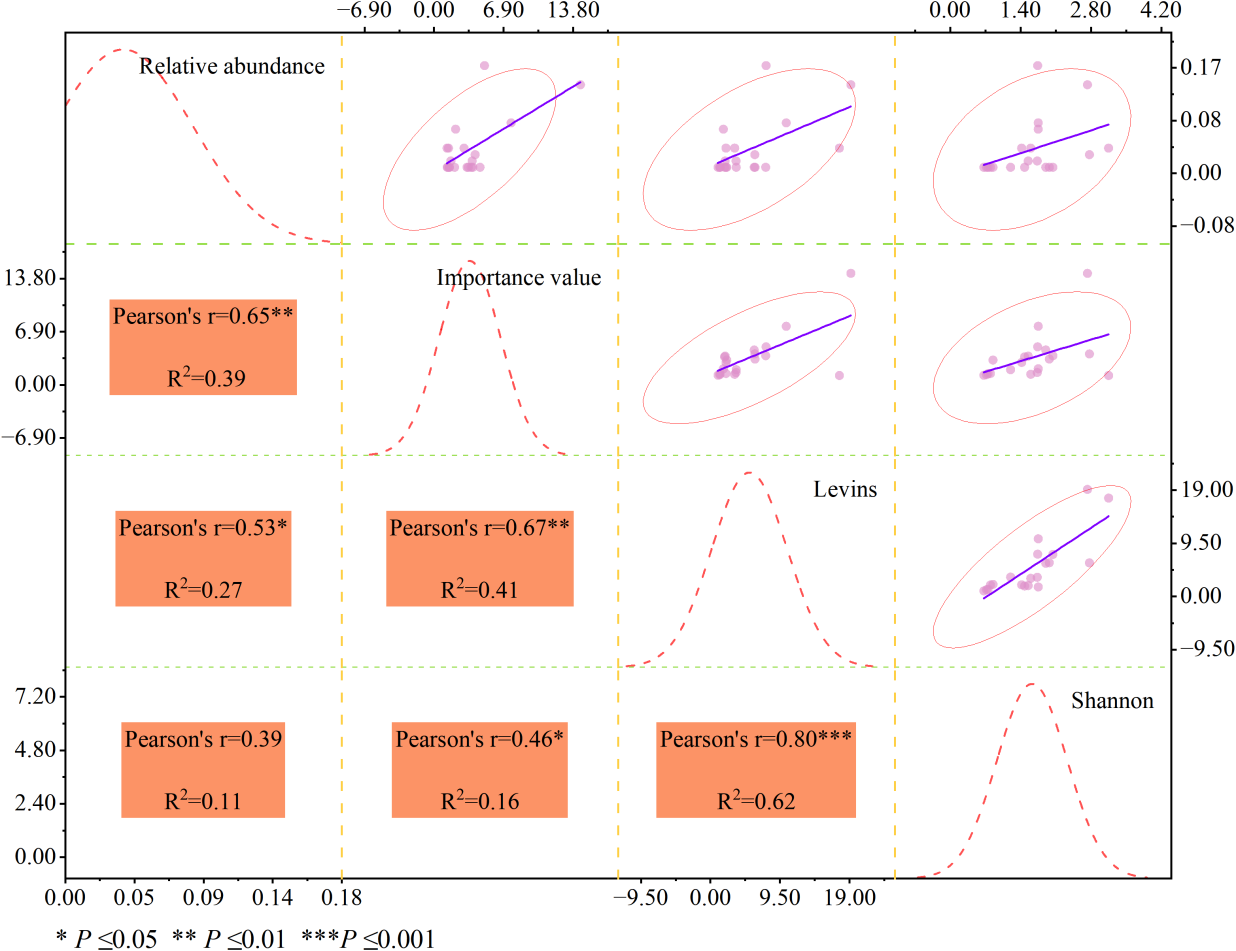
Relationships between species dominance characteristics and niche breadth indices for the 20 dominant herbaceous species in alpine semi-arid river valleys.

Correlation analysis was performed to quantify the interrelationships among relative abundance, importance value, and two classic niche breadth indices (Levins and Shannon) of dominant species in the herb layer, with the results shown in **Figure 2**. The Levins and Shannon niche breadth indices, which characterize the resource utilization range of species, showed an extremely significant positive correlation (Pearson’s r=0.80, *P* ≤0.001), with a coefficient of determination *R*²=0.62. This result indicated that the variation trends of the two niche breadth indices were highly consistent, both of which could effectively reflect the habitat adaptation capacity and resource utilization strategy of dominant species, which was consistent with the niche breadth differentiation characteristics described above.The importance value, a comprehensive indicator of species ecological dominance, had a more pronounced synergistic relationship with niche breadth. Specifically, the importance value presented an extremely significant positive correlation with the Levins niche breadth index (Pearson’s r=0.67, *P* ≤0.01, *R*²=0.41), and a significant positive correlation with the Shannon niche breadth index (Pearson’s r=0.46, *P* ≤0.05, *R*²=0.16). This demonstrated that species with wider niche breadths generally had higher ecological dominance in the community. The wider the resource utilization spectrum of a species, the stronger its adaptability to heterogeneous habitats, and thus the more prominent its dominant position in the herb community.In contrast, relative abundance, which reflects the quantitative proportion of species in the community, showed a weaker and more complex correlation with niche breadth. The relative abundance only had a significant positive correlation with the Levins niche breadth index (Pearson’s r=0.53, *P* ≤0.05, *R*²=0.27), while its positive correlation with the Shannon niche breadth index was not statistically significant (Pearson’s r=0.39, *P >*0.05, *R*²=0.11). This result further confirmed that the quantitative dominance of species was not completely synchronized with their niche breadth: some species with high relative abundance did not have a wide resource utilization range, which was mainly attributed to their strong habitat specialization and high dependence on specific microhabitats, rather than an extensive resource utilization strategy.Meanwhile, the relative abundance of dominant species showed an extremely significant positive correlation with the importance value (Pearson’s r=0.65, *P* ≤0.01, *R*²=0.39), indicating that the quantitative proportion of species was an important component of their comprehensive ecological dominance, but not the sole determining factor. The community status of species was jointly shaped by their quantitative characteristics, resource utilization capacity and heterogeneous habitat adaptation potential.

### 3.3.1 Analysis of niche overlap index

**Figure 3:**
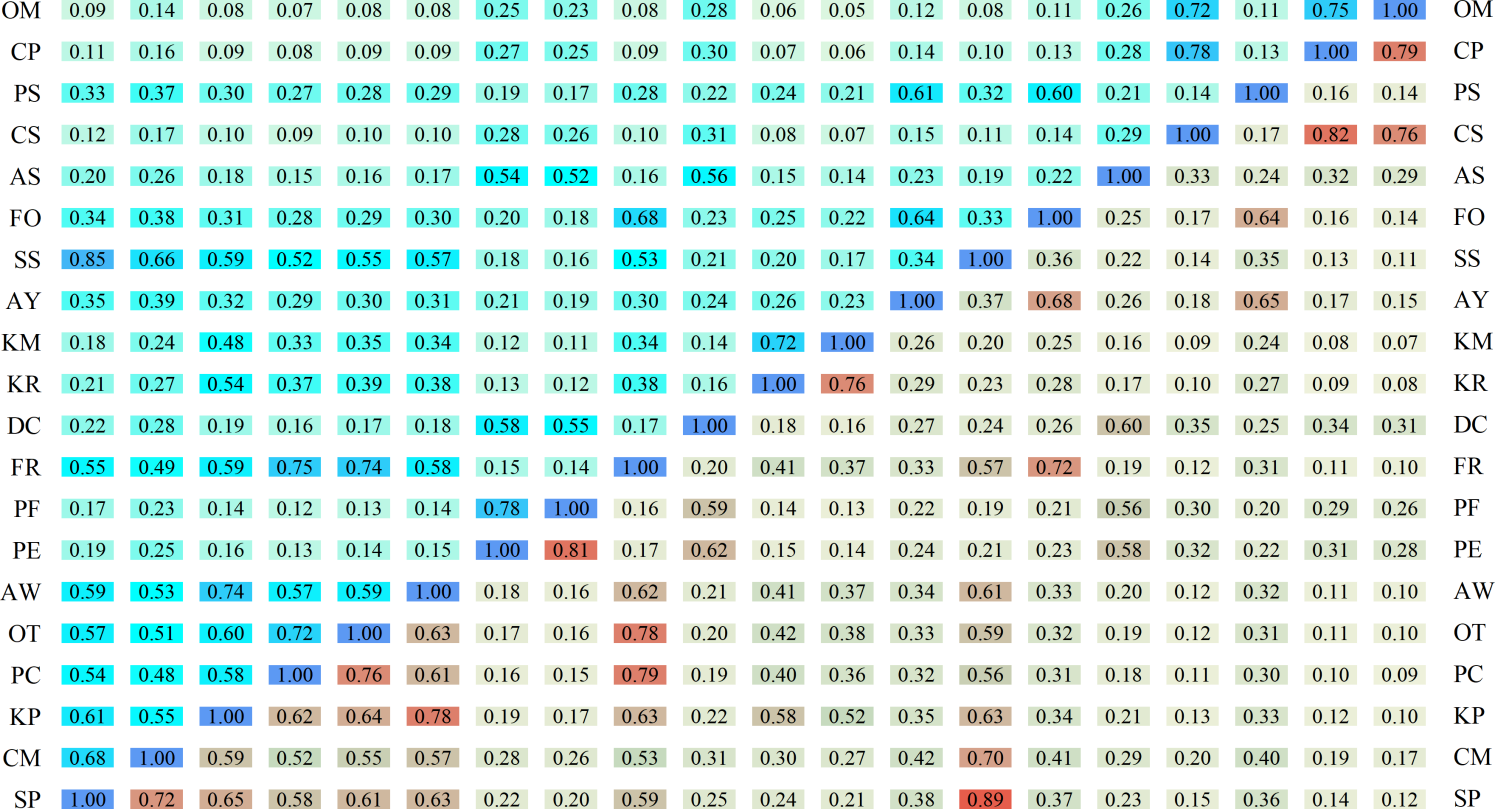
Niche overlap indices (upper-left corner) and similarity indices (lower-right corner) of dominant species in the herb layer.

The niche overlap and niche similarity among dominant species in the herb layer showed significant differentiation. The Pianka niche overlap index ranged from 0.05 to 0.89, and the Schoener niche similarity index ranged from 0.05 to 0.85, with highly consistent variation trends between the two indices, indicating a strong correlation between resource utilization overlap and ecological strategy similarity among species.The highest niche overlap and similarity were observed between *S. purpurea* and *S. subsessiliflora*, which are congeneric species with the widest Levins niche breadth, indicating that these two species had extremely high overlap in resource utilization and similar habitat adaptation strategies, with the strongest potential interspecific competition. High niche overlap was also found between congeneric species pairs, including *P. centrasiaticum* and *P. flaccidum*, *K. royleana*and *K. macrantha*, *C. sagaensis* and *C. praeclara*, reflecting the ecological niche convergence of phylogenetically closely related species.Species with wide niche breadths showed generally high niche overlap with other species. *S. purpurea*, the core dominant species with the largest niche breadth, had an average overlap index of 0.42 with all other species, and *S. subsessiliflora* had an average overlap index of 0.40, indicating that these two widely adapted species had extensive resource utilization overlap with most species in the community. In contrast, species with narrow niche breadths had extremely low interspecific overlap: *O. microphylla*, *C. praeclara* and *C. sagaensis*, which had the narrowest niche breadths, had average overlap indices below 0.20 with other species, indicating their high habitat specialization and sufficient resource differentiation from other species, with minimal interspecific competition. Notably, *K. pygmaea*, which had the highest relative abundance, showed high niche overlap with *A. wellbyi*, but low overlap with narrow-niche species, suggesting that its numerical dominance was mainly derived from the efficient utilization of specific resources, rather than extensive competition with all species. *Artemisia younghusbandii*, *Festuca ovina* and *Polygonum sibiricum* Laxm. showed high niche similarity with each other, indicating that these species had similar habitat preferences and formed a coordinated species group in the community(**Figure 3**).

### 3.4 Inter-species connectivity analysis

#### 3.4.1 Overall connectivity analysis

The *VR* value of the herbaceous layer in the study area is 1.032, with *VR* > 1, indicating a positive association between the plant layers. A total of 109 plots were set up in the study area, with the statistical test value *W* being 112.49. The *χ*² critical values are (85.05, 134.66). The test statistic *W* falls within the defined interval between *χ*²0.05_(N)_ and *χ*²0.95_(N)_. The overall association is not significant.

#### 3.4.2 Connectivity coefficient analysis

**Figure 4:**
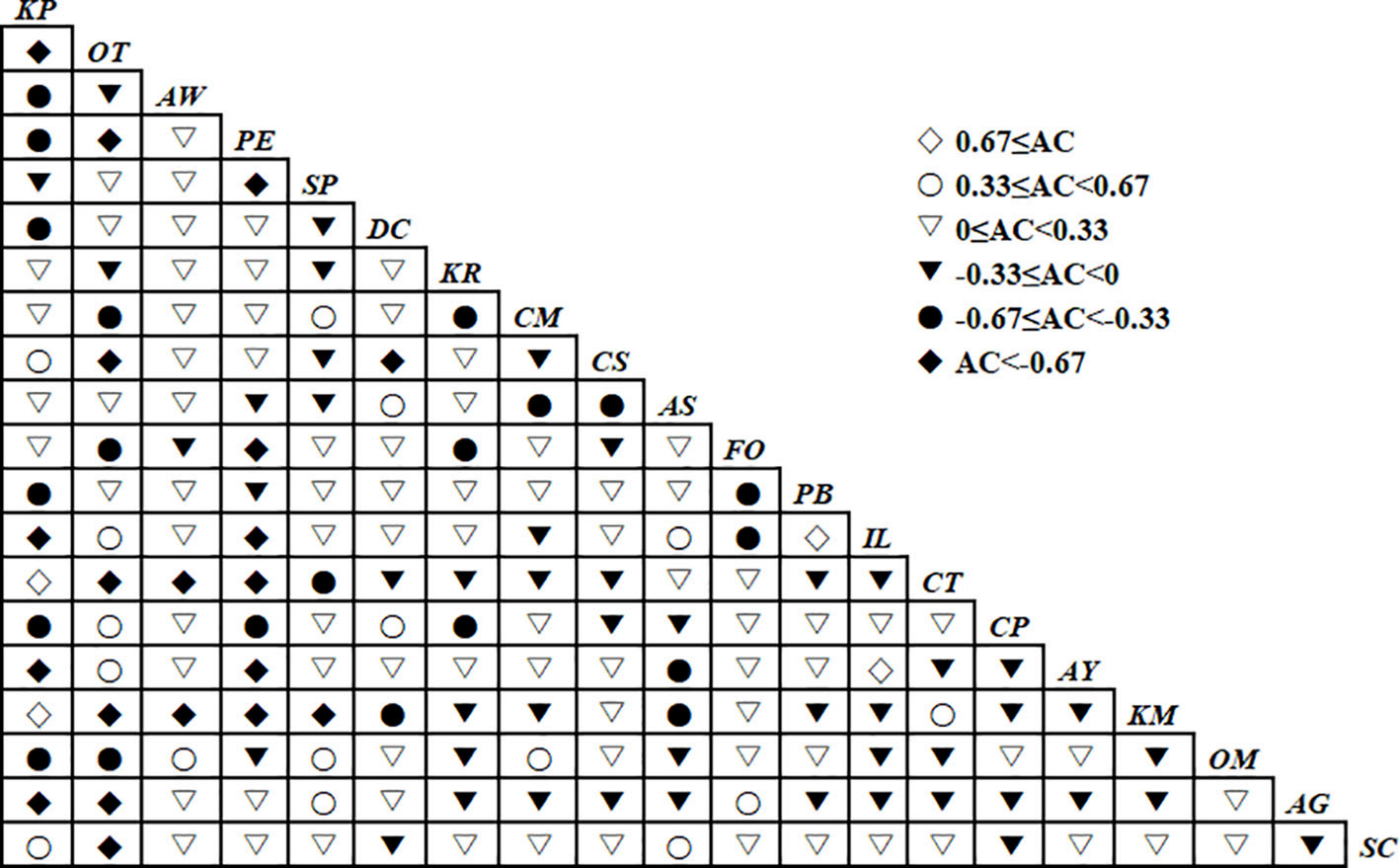
Semi-matrix diagram showing the pairwise interspecific association coefficientresults of the 20 dominant herbaceous species in the herb layer.

The association coefficient(*AC*) analysis results for the herbaceous layer in the study area show that 52.63% of species pairs (100 pairs) have an *AC* coefficient greater than 0. Among them, 82 species pairs fall within the range of 0 ≤ *AC* < 0.33, accounting for 43.16% of the total pairs, such as *K.royleana* and *A. younghusbandii*, *C. moorcroftii* and *A. wellbyi*, etc. There are 16 species pairs within the range of 0.33 ≤ *AC* < 0.67, accounting for 8.42% of the total species pairs, such a*s K. pygmaea* and *P.sibiricum*, *S. purpurea* and *F. rubra*, etc. There are 2 species pairs within the range of 0.67 ≤ *AC*, accounting for 1.05% of the total species pairs, namely *A. younghusbandii* and *A. strictus*, *S. purpurea* and *S. subsessiliflora*, *S. purpurea* and *C. sagaensis*. 47.37% of species pairs (90pairs) have an *AC* association coefficient less than 0. There are 48species pairs within the range of -0.33 ≤ *AC* < 0, accounting for 25.26% of the total species pairs, such as *O. thoroldii* and *A.wellbyi*, *P. centrasiaticum* and *D.chraderiana*, etc. There are 21 species pairs within the range of -0.67 ≤ *AC* < -0.33, accounting for 11.05% of the total species pairs, such as *P.centrasiaticum* and *F. ovina*, *O. thoroldii* and *S.subsessiliflora*, etc. There are 21 species pairs within the range of *AC*< -0.67, accounting for 11.05% of the total species pairs, such as *S. purpurea* and *C. moorcroftii*, *Iris K. pygmaea* and *S. subsessiliflora*, etc. Due to uneven species distribution in some plots, there is a tendency for a = 0, which increases the number of species pairs falling within the range of *AC* < -0.67. However, the trends of positive and negative associations, as well as species pairs with higher association coefficients, are generally consistent with the chi-square test results, and both can serve as a good validation. To further validate the interspecific associations of species in the study area, Ochiai index and community stability analyses will be conducted (**Figure 4**).

#### 3.4.3 Ochiai index analysis

**Figure 5:**
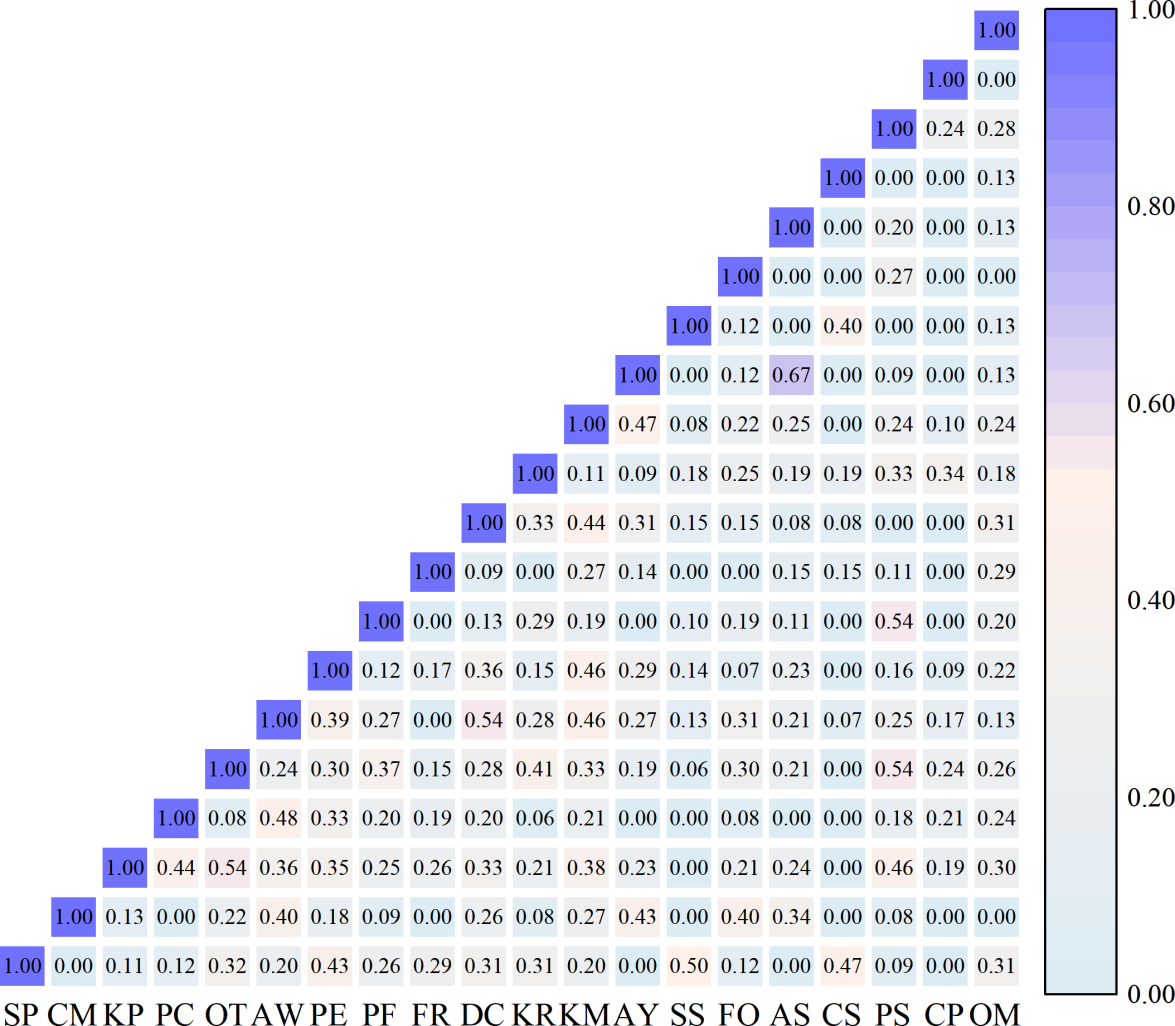
Ochiai association index analysis among 190 pairwise species combinations of the 20 dominant herbaceous species.

The analysis results of the *OI* for the herbaceous layer in the study area show that the mean value of the *OI* approximately 0.18, indicating a low compositional similarity among species. Among them, 23.68% of species pairs (45 pairs) have an OI value of 0, representing no co-occurrence between the paired species. 45.26% of species pairs (86 pairs) fall within the range of 0.00 <*OI ≤* 0.25, which is consistent with the results of the AC association test, further confirming the independence and differentiation of interspecific associations in the herbaceous layer. 2.11% of species pairs (4 pairs) fall within the range of 0.25 <*OI* ≤0.50, accounting for 2.11% of the total species pairs. 0.53% of species pairs (1 pair) fall within the range of 0.50 <*OI*≤ 0.67, with the maximum *OI* value of 0.67 recorded between *A. younghusbandii* and *A. strictus*. The overall results indicate that the ecological association between species is weak, with significant differentiation in resource utilization strategies and niche characteristics among co-occurring species (**Figure 5**).

#### 3.4.4 Community stability analysis

**Figure 6:**
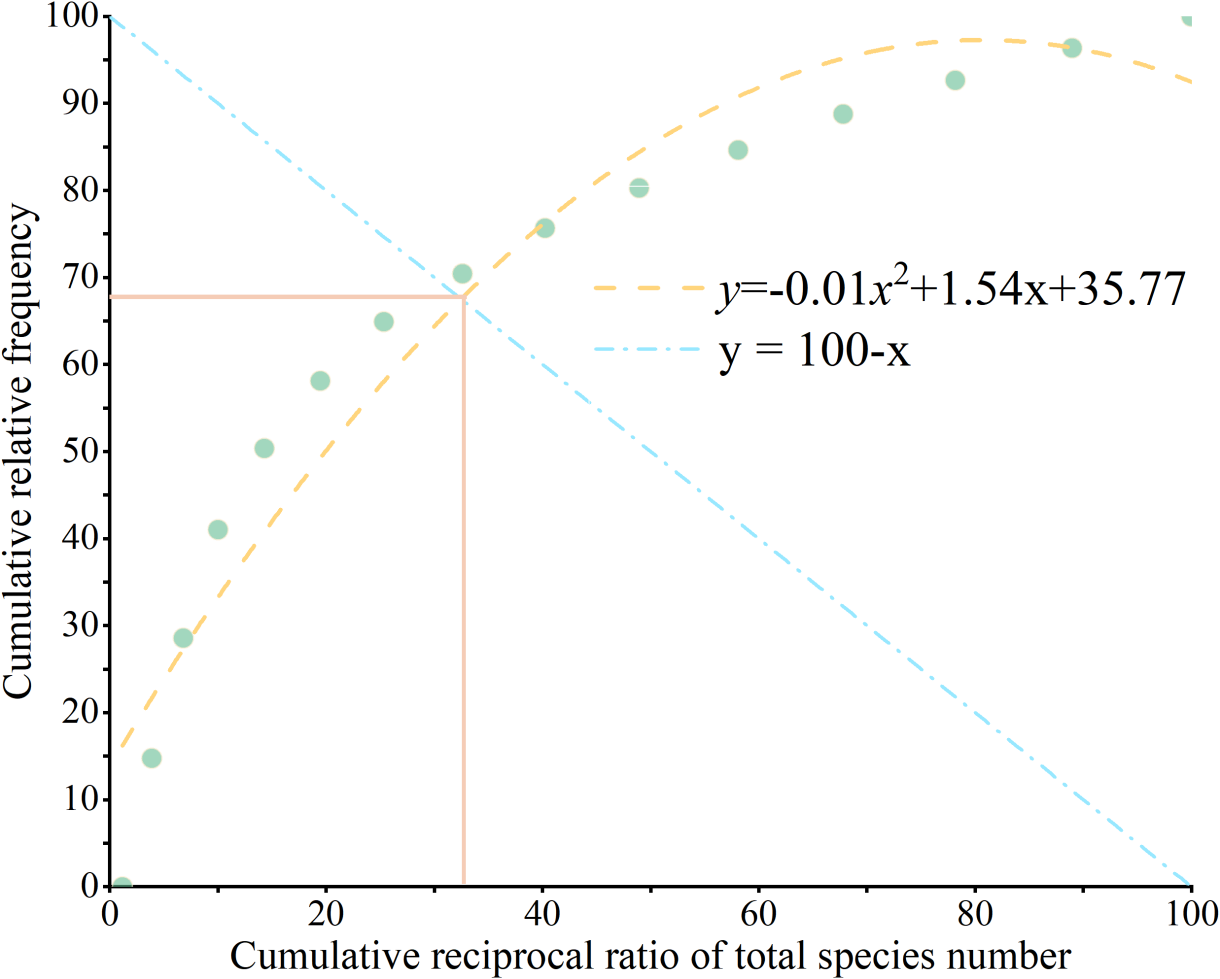
Herb layer community atability analysis.

To further quantify the stability of the herbaceous community in the study area, we adopted the Godron stability analysis method, a classic and widely used approach to evaluate plant community stability by measuring the deviation between the actual fitted curve of species cumulative frequency and the theoretically ideal stable state. The stability reference line representing the ideal stable community was defined as *y* = 100-*x*, while the nonlinear fitting equation established based on the cumulative relative frequency of dominant species in the herbaceous layer was *y* = -0.01*x*² + 1.54*x* + 35.77. The two curves intersected at the coordinate (33.68, 68.73), and the Euclidean distance between this intersection and the theoretical optimal stable point (20, 80) was 17.22. This relatively large Euclidean distance indicated a high deviation of the actual community dynamic trajectory from the ideal stable state. Combined with the previous results of the Ochiai association coefficient and AC association coefficient, which showed generally weak interspecific associations and significant niche differentiation among dominant species, the stability analysis results consistently demonstrated that the herbaceous layer in the study area was generally in an unstable state (**Figure 6**).

## 4 Discussion

### 4.1 Niche characteristics and their adaptation mechanisms to heterogeneous habitats

Niche differentiation is the core of community assembly and species coexistence, and its expression in extreme alpine habitats is a key focus of ecological research. Our results showed that the dominant herbaceous species in the study area formed significant niche differentiation, with a clear divergence between broad-niche generalists and narrow-niche specialists, which is a directed adaptation to the extreme heterogeneous habitats of alpine semi-arid river valleys (Carboni et al., 2016; Marshall and Elliott, 1997). *S.purpurea* and *S.subsessiliflora* with the highest Levins and Shannon indices are typical generalists, with a broad tolerance range to the continuous hydrothermal and soil gradients of the valley system; while *O.microphylla*, *C.praeclara* and *C.sagaensis* with extremely narrow niche breadth are typical specialists, relying on high-efficiency utilization of specific microhabitats to avoid interspecific competition. This differentiation pattern is consistent with related studies in alpine meadows of the Qinghai-Xizang Plateau (Wang et al., 2024), and the more pronounced differentiation in this study is mainly attributed to the more complex multi-dimensional resource gradient formed by the alpine gorge landform and continuous valley habitat sequence.Further analysis found that species ecological dominance (importance value) was significantly positively correlated with niche breadth, but relative abundance was not completely synchronized with niche width, reflecting differentiated survival strategies of alpine plants. For example, *O.thoroldii* showed a “high abundance-high importance value-narrow niche” pattern, maintaining population dominance through specific microhabitat specialization, which is consistent with the conclusion that dominant species in alpine desert grasslands maintain populations via habitat specificity (Liu et al., 2025). In contrast, *K. pygmaea* with the highest relative abundance only had moderate niche breadth, indicating its numerical dominance was derived from efficient utilization of specific resources rather than extensive resource competition, which complements the research on the relationship between niche breadth and species dominance in heterogeneous environments (Li et al., 2024).In terms of niche overlap, the overall low overlap level among species is direct evidence of fine-scale resource partitioning in the community, which is significantly lower than that of alpine temperate grasslands on the Qinghai-Xizang Plateau (Wu et al., 2022), and similar to studies in the Altai region with similar climatic conditions (Su et al., 2024). Even congeneric species with high niche overlap did not show intense competitive exclusion, which is speculated to be related to their differentiation in phenology or microhabitat preference, and this phenomenon conforms to Chesson’s theory that resource differentiation reduces competition and promotes species coexistence (Chesson and Shea, 2002). This refined niche differentiation is not only a species-level adaptive strategy to alpine stress, but also optimizes the allocation of limited resources in the community, which is the key mechanism for maintaining ecosystem functions under resource scarcity.

### 4.2 Interspecific association characteristics and community assembly mechanisms

The herbaceous layer in the study area showed a weak positive and non-significant overall interspecific association, with most species pairs having no significant association, presenting a loose community structure. This is not a sign of community instability, but a key adaptive strategy for alpine plants to reduce survival risks in extreme environments, and also directly reflects the loose assembly characteristics of alpine plant communities (Graae et al., 2018; Zanzottera et al., 2020). Among them, significant positive associations were mainly concentrated among broad-niche generalists, while significant negative associations only occurred between species with high niche overlap and similar resource requirements, which is consistent with the core prediction of classical niche theory.The formation of this weak association pattern is mainly driven by two factors. First, in high-altitude extreme environments, energy is extremely scarce, and species need to allocate most of their energy to cope with abiotic stresses such as low temperature, strong UV radiation and seasonal drought, resulting in extremely limited energy available for interspecific cooperation or competition (Parsons, 2005; Sømme, 1989). Even species with complementary niches can only form minimal nutrient exchange, making it difficult to establish stable and strong interdependent relationships (Liu et al., 2024; Sara et al., 2025), which directly limits the formation of strong positive associations. Second, the alpine gorge topography leads to fragmented and isolated microhabitats, and different species are confined to their respective suitable microhabitats, resulting in an extremely low probability of spatial co-occurrence between species (Strong et al., 2021). This not only reduces the opportunity for interspecific cooperation, but also weakens the intensity of competitive exclusion, which explains the scarcity of significant negative associations, and is highly consistent with the theory that negative associations are strongest when niche overlap is moderate (Papuga et al., 2018), as well as the adaptation strategy that alpine species tend to tolerate moderate competition rather than intense exclusion under environmental stress (Xu et al., 2016).From the perspective of ecological function, this loose structure with weak interspecific associations endows the community with unique disturbance resistance in extreme environments (Oliver et al., 2015). On the one hand, weak interspecific interactions can effectively isolate the propagation of population fluctuations caused by local environmental disturbances, preventing the overall community structure from being affected by local disturbances, which is the fluctuation isolation effect of loose community structure (Sousa, 1984; Altermatt et al., 2011). On the other hand, the significant niche differentiation formed by habitat fragmentation retains the potential for vacant niche compensation. When a species declines due to disturbance, the vacant resources can be quickly filled by other species with complementary niches, effectively preventing the loss of key community functions (Nour et al., 1997; Swihart et al., 2003). This characteristic of “local fluctuations not propagating to the whole system” is a key adaptive advantage of alpine herbaceous communities compared with low-altitude communities.

### 4.3 Community stability characteristics and their influencing factors

Godron stability analysis showed that the herbaceous community in the study area was in an insufficiently stable state, with a Euclidean distance of 17.22 from the theoretical optimal stable point (20, 80), which is consistent with the characteristics of weak interspecific associations and insufficient functional support from niche differentiation (Zeng et al., 2025). The core underlying causes of insufficient stability are the dispersed dominance of dominant species, the limited number of broad-niche generalists, and the generally weak interspecific associations (Brown and Dyer, 1982; Lin et al., 2025). Specifically, the small difference in importance values among the top 20 dominant species leads to the lack of core dominant species with strong regulatory capacity, making it difficult for the community to quickly recover after disturbance; the scarcity of broad-niche species limits the community’s ability to fill vacant niches under environmental changes; and weak interspecific associations lead to the lack of a complete functional complementarity network, weakening the community’s buffering effect against external disturbances (Amyntas et al., 2023; Hou et al., 2023; Xiang et al., 2024).It is worth noting that the community presents a “low structural stability - high disturbance resistance” paradox, which is a unique adaptive strategy of herbaceous communities in alpine semi-arid river valleys. On the one hand, high niche differentiation reduces the dependence between species, making the fluctuation of a single species have little impact on the whole community, which is consistent with the “loose structure resilience to disturbance” hypothesis (Pastore et al., 2021); on the other hand, weak interspecific associations make the community lack synergistic protection mechanisms, and it is difficult for other species to provide support for stressed broad-niche species through positive interactions (Lin et al., 2025). This paradox is similar to the “resilient stability” characteristics of riparian plant communities, but the community recovery ability in this study area is weaker due to the high-altitude environment limiting species migration and colonization rates (Jin et al., 2025).In addition, the regional climate and topographic characteristics are the fundamental background shaping community stability. The plateau temperate semi-arid monsoon climate with large diurnal temperature variation and seasonal drought, as well as the dramatic topographic relief of alpine gorges, lead to severe fluctuations in resource availability, making it difficult for species to form stable coexistence strategies (Liu et al., 2025). Meanwhile, dominant species need to allocate more energy to survival rather than interspecific interactions and community construction under multiple stresses (Billings and Mooney, 1968), which is the root cause of low community stability. Compared with other alpine regions around the world, the niche differentiation degree of the community in this study area is higher than that in the European Alps (Choler and Michalet, 2002), but the stability is lower than that in the Andean alpine meadows (Cuesta et al., 2023; Tovar et al., 2020), which may be related to the relatively short uplift history of the Qinghai-Xizang Plateau and the insufficiently formed stable coexistence mechanisms of species.

## 5 Conclusion

Based on 5-year consecutive field monitoring in the alpine semi-arid river valleys of the eastern Qinghai-Xizang Plateau, we systematically analyzed the niche characteristics, interspecific associations and community stability of dominant herbaceous species, with the following core conclusions:Significant niche differentiation is the core strategy for herbaceous species to avoid competitive exclusion and achieve stable coexistence. Dominant species formed a clear divergence between broad-niche generalists and narrow-niche specialists, with low overall niche overlap and a fine-scale resource partitioning pattern adapted to local heterogeneous habitats.The herb layer presented an overall non-significant weak positive interspecific association and a loose community assembly pattern; significant positive associations were mainly concentrated among broad-niche generalists, while significant negative associations only occurred between species with highly similar resource requirements.The herbaceous community in the study area showed insufficient stability, mainly constrained by dispersed dominance of dominant species, limited number of broad-niche species, and generally weak interspecific associations.This study has limitations in monitoring duration, extreme microhabitat coverage and underlying driving mechanism clarification, and future research will combine long-term fixed monitoring, functional trait analysis and *in-situ* controlled experiments to further reveal the assembly mechanisms and long-term dynamics of alpine herbaceous communities under climate change.Our findings fill the key research gap in the ecology of herb layers in the eastern Qinghai-Xizang Plateau’s alpine semi-arid valleys, and provide a scientific basis for the conservation and restoration of alpine ecosystems.

## Data Availability

The original contributions presented in the study are included in the article/supplementary material. Further inquiries can be directed to the corresponding authors.
